# Consolidative Radiotherapy after Complete Remission following R-CHOP Immunochemotherapy in Stage III–IV Diffuse Large B-Cell Lymphoma Patients: A Systematic Review and Meta-Analysis

**DOI:** 10.3390/cancers15153940

**Published:** 2023-08-02

**Authors:** Kyu-Hye Choi, Seung-Jae Lee, So-Hwa Mun, Jin-Ho Song, Byung-Ock Choi

**Affiliations:** 1 Department of Radiation Oncology, Seoul St. Mary’s Hospital, College of Medicine, The Catholic University of Korea, Seoul 06591, Republic of Korea; kyuhye@gmail.com (K.-H.C.);; 2 Medical Library, The Catholic University of Korea, Seoul 06591, Republic of Korea

**Keywords:** diffuse large B-cell lymphoma, advanced stage, consolidative radiotherapy

## Abstract

**Simple Summary:**

The present systematic review and meta-analysis aimed to clarify the role of consolidative RT among patients with advanced-stage DLBCL who achieved complete remission after rituximab-based immunochemotherapy (R-CHOP). Six retrospective studies involving 319 individuals who underwent R-CHOP consolidative RT and 494 individuals who did not undergo consolidative RT were included. Consolidative RT was associated with superior overall survival (HR: 2.01; 95% CI: 1.30–3.12; *p* = 0.002) and disease-free survival (HR: 2.18; 95% CI: 1.47–3.24; *p* < 0.0001). These results showed that consolidative RT is superior to no RT in terms of both overall survival and disease-free survival for patients with advanced-stage DLBCL. Further investigation is warranted to determine the optimal radiation fields and the appropriate indications for consolidative RT in patients with advanced-stage DLBCL in the rituximab era.

**Abstract:**

Patients with diffuse large B-cell lymphoma (DLBCL) are treated with rituximab in combination with cyclophosphamide, doxorubicin, vincristine, and prednisone (R-CHOP). The role of consolidative radiation therapy (RT) remains unclear among patients with advanced DLBCL who achieved complete remission (CR) after R-CHOP immunochemotherapy. The current systematic review and meta-analysis aimed to clarify the role of consolidative RT among these patients. The MEDLINE, Embase, and Cochrane Library databases were searched for studies comparing RT to no RT following CR after R-CHOP immunochemotherapy in Ann Arbor stage III–IV DLBCL patients. Overall survival (OS) was the primary endpoint, and disease-free survival (DFS) was the secondary endpoint. Hazard ratios (HRs) and 95% confidence intervals (CIs) were calculated to assess the primary and secondary outcomes. Review Manager (version 5.4) was used to analyze the data. Six retrospective studies involving 813 patients who received R-CHOP ± consolidative RT were identified. OS was higher in the consolidative RT group, with an HR of 2.01 and a 95% CI of 1.30 to 3.12 (*p* = 0.002). DFS was also higher in the RT group, with an HR of 2.18 and a 95% CI of 1.47 to 3.24 (*p* < 0.0001). The results suggested that consolidative RT improved OS and DFS compared to no RT among advanced-stage DLBCL patients. Further research is needed to determine the optimal radiation fields and the appropriate indications for consolidative RT for advanced-stage DLBCL patients in the rituximab era.

## 1. Introduction

Diffuse large B-cell lymphoma (DLBCL) is the most prevalent type of non-Hodgkin’s lymphoma, characterized by aggressive growth and frequently presenting in advanced stage III–IV [1]. Recently, rituximab, a monoclonal antibody against CD20, has increased the survival rate of DLBCL patients, and rituximab plus CHOP (cyclophosphamide, doxorubicin, vincristine, prednisone; R-CHOP) has become a standard treatment [2,3]. However, the ten-year survival rate for advanced-stage DLBCL patients remains as low as 40% [4]. In addition, the high relapse rate and limited success of salvage treatment underscore the importance of adjuvant treatment.

Consolidative radiation therapy (RT) is commonly used after chemotherapy to reduce recurrence in patients with limited-stage DLBCL [5]. Previous research has demonstrated that consolidative RT in patients with early stage DLBCL can improve local control (LC), progression-free survival (PFS), and overall survival (OS). The role of consolidative RT in the advanced stages of DLBCL, however, remains unclear. Retrospective studies have shown conflicting results, with some indicating that patients who received RT at the bulky site exhibited improved survival rates [6,7]. Nonetheless, there have been many controversies among the results.

Despite the potential of consolidative RT in advanced DLBCL to improve LC and survival, clinical implementation is rare due to concerns about radiation-related toxicity. Consequently, the effectiveness of this approach in advanced-stage DLBCL is still unclear. This study aimed to aggregate and analyze the previously published data to establish the evidence and feasibility of consolidative RT in the management of advanced-stage DLBCL.

## 2. Materials and Methods

### 2.1. Searching and Selection

The current study aimed to address the PICO question, “Does consolidative RT after complete remission following R-CHOP improve clinical outcomes in advanced-stage diffuse large B-cell lymphoma patients?” A systematic search of the Medline, EMBASE, and Cochrane Library databases was conducted for articles published until 22 October 2022. The inclusion criteria for the study were as follows: patients diagnosed with DLBCL Ann Arbor stage III–IV who exhibited complete remission (CR) after R-CHOP immunochemotherapy and who were treated with or without consolidative RT. The PRISMA guidelines were followed, and the recommendations outlined in the Cochrane Handbook version 6.3 were adhered to [8]. This study was registered in PROSPERO (ID: CRD42023414176).

### 2.2. Data Collection and Quality Assessment

This study focused on a cohort of patients with Ann Arbor stage III–IV DLBCL who achieved CR after R-CHOP immunochemotherapy. The review investigated adjuvant interventions to improve OS and disease-free survival (DFS) rates. The adjuvant intervention to be compared was consolidative RT. The primary outcome was the five-year OS rate, and the secondary outcome was the five-year DFS rate, including the progression-free or event-free survival rate. Treatment-related toxicities were also assessed. Data were independently extracted by two reviewers (KHC and SJL), and any discrepancies were resolved through consensus. Data were collected using a pretested spreadsheet, which included variables such as sample size for intervention and control groups, demographic characteristics, intervention details, outcome measures, the first author’s name, publication year, and follow-up period.

Two independent reviewers (KHC and SHM) assessed the quality of the selected studies. For nonrandomized and observational studies, the Newcastle-Ottawa Scale (NOS) was used to score the quality of each study [9]. The studies were classified as high quality if they received more than 8 points, medium quality for scores between 6 and 7 points, and low quality for scores below 6 points. Discrepancies between the two reviewers were resolved through discussion with the corresponding author (BOC).

### 2.3. Statistical Analysis

Meta-analyses were conducted using Review Manager 5.4 (RevMan, The Cochrane Collaboration, Oxford, UK) statistical software. The primary and secondary outcome measures were analyzed using hazard ratios (HRs) and 95% confidence intervals (CIs). Heterogeneity in the included studies was assessed using the I^2^ statistic and chi-square test. Significant heterogeneity was determined when a *p* value of < 0.1 and an I^2^ value of >50% were found. The random effects model was employed for the meta-analysis, and the association between consolidative RT and either OS or DFS was derived as a weighted average of study-specific estimates of the HR using inverse variance weights.

## 3. Results

**Figure 1:**
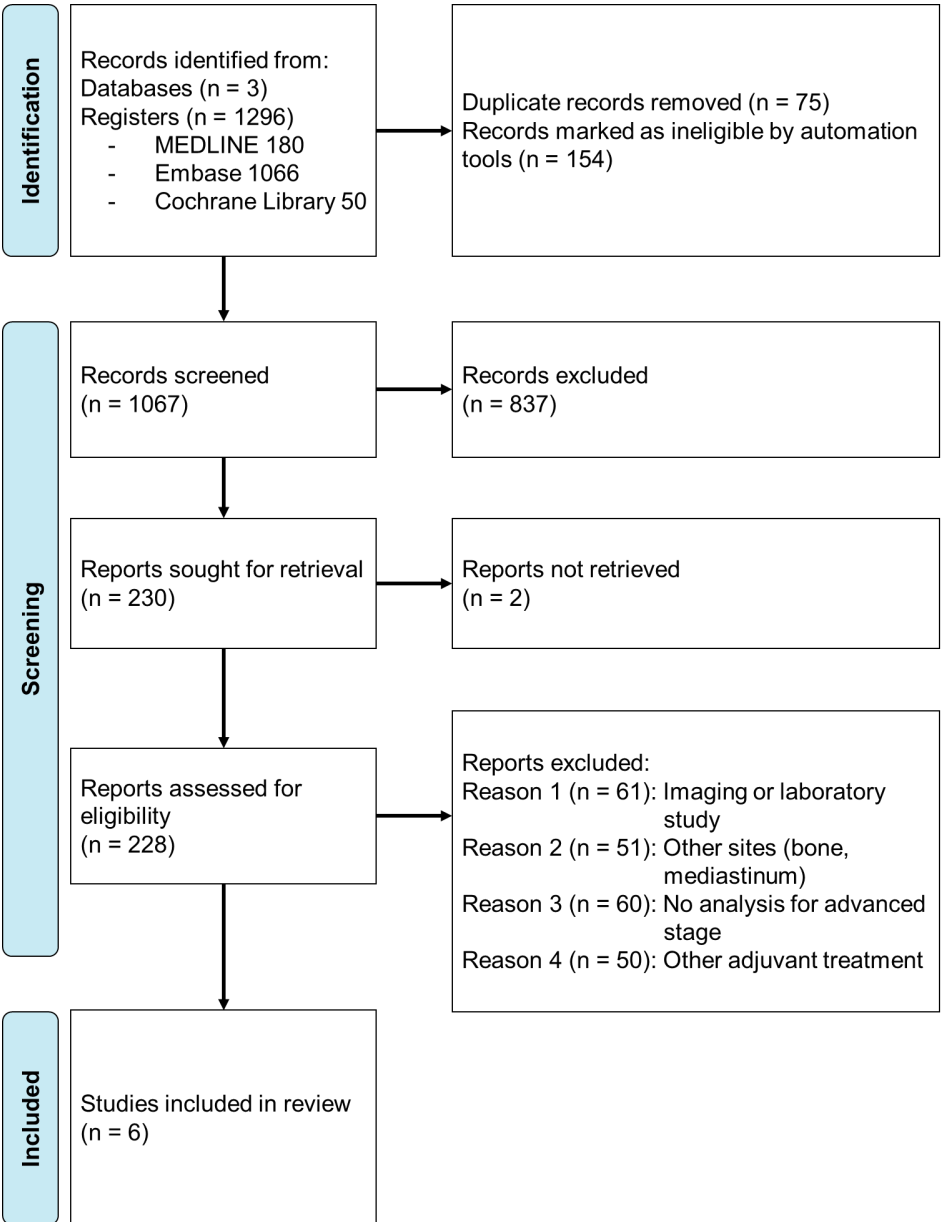
PRISMA flow diagram.

Initially, 1296 studies were found in the Medline, EMBASE, and Cochrane Library databases. After removing duplicates and ineligible records, 1067 records were screened. Of those, 228 studies were subjected to full-text review to exclude irrelevant subjects or those that did not fully meet the inclusion criteria. Finally, six studies were included in the meta-analysis, which comprised 813 patients in total. Among them, 319 patients underwent consolidative RT, while 494 patients did not receive adjuvant treatment. The retrieval process is summarized in Figure 1.

All six studies included in the meta-analysis were original retrospective articles [6,7,10,11,12,13]. Five studies analyzed DLBCL patients with Ann Arbor stage III–IV disease, and subgroup analysis according to stages III to IV was performed in one study [10]. Comparisons of demographics between the two groups were conducted, while two studies used propensity score matching [10,12]. The characteristics of the included studies are summarized in Table 1. Quality assessment was conducted using the NOS. Based on the NOS measurements, three studies were classified as high quality, and the other three were considered medium quality. The scoring sheet can be found in Supplementary Materials Table S1.

The median radiation dose ranged from 25 to 30 Gy, and the median follow-up period ranged from 33 to 61 months. All studies statistically analyzed the differences in OS, and none of them showed statistically significant improvement in OS for consolidative RT. Among the five studies that provided the statistical significance of the DFS analysis, three studies showed a significant difference, while the others did not. The clinical results of each study are shown in Table 2.

**Figure 2:**
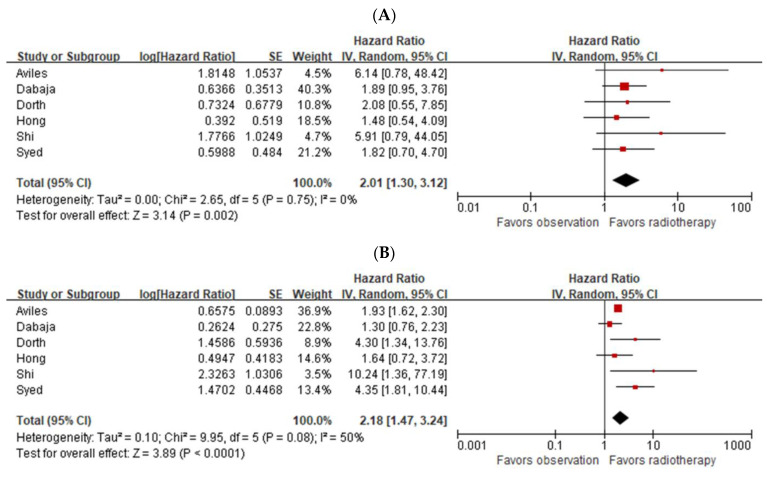
Forest plots of overall survival (**A**) and disease-free survival (**B**) [6,7,10,11,12,13]. Each study is represented by a box (point estimate) and a horizontal line (95% CI). The size of the box reflects the weight of the study, with larger boxes indicating studies that contributed more data and provided greater weight. The combined pooled effect derived from the included studies is depicted as a diamond located below the individual studies.

The meta-analysis using the generic inverse variance method revealed a two-fold increase in the five-year OS rate among patients who received consolidative RT compared to those who did not (HR: 2.01; 95% CI: 1.30–3.12; *p* = 0.002), with no heterogeneity among the studies (I^2^ = 0%; *p* = 0.75). A meta-analysis of the 5-year DFS demonstrated an HR of 2.18 (95% CI: 1.47–3.24; *p* < 0.0001), and there was moderate heterogeneity among studies (I^2^ = 50%; *p* = 0.08). The forest plots of OS and DFS are presented in Figure 2. Each study is represented by a box (point estimate) and a horizontal line (95% CI). The size of the box reflects the weight of the study, with larger boxes indicating studies that contributed more data and provided greater weight. The combined pooled effect derived from the included studies is depicted as a diamond located below the individual studies. The funnel plots of OS and DFS according to the analyzed studies were used to assess publication bias and are shown in Supplementary Materials Figure S1. There was no obvious asymmetry identified in the funnel plot shape.

The radiation fields of the three studies focused exclusively on high-risk regions, such as bulky sites, bony lesions, or head and neck areas [6,7,12]. In a study conducted by Shi et al. [6], RT was administered to all initially presenting sites in 50% of the cases (*n* = 7), which included the involved lymph nodes and their regions or involved organs and the corresponding draining lymphatics. In the remaining cases, RT was limited to treating bulky disease due to toxicity concerns. Twenty-one patients (19.1%) experienced treatment failure at both the initial presentation site and distant site, and this outcome was observed exclusively in the R-CHOP alone group (21.9% vs. 0% in the R-CHOP + RT group). LC demonstrated a strong correlation with the distant control, as only a few patients experienced distant treatment failure without having experienced local failure at the site treated with R-CHOP alone.

Aviles et al. [7] performed radiation to initially bulky areas (>10 cm) without using modern RT techniques. Notably, there was no local recurrence in the RT group, and the R-CHOP alone group observed both local and distant recurrences (*n* = 36) more frequently than distant recurrence alone (*n* = 5). Hong et al. [12] delivered RT to initially bulky areas (≥5 cm), head and neck lesions, and testicular and bone lesions. None of the patients received RT at all the involved sites. The most frequent site of local recurrence was observed in the head and neck, affecting 10 patients (50.0%). Among the 14 patients who experienced local recurrence, 6 (42.9%) patients had local recurrence in the head and neck lesions. The second most common site of local recurrence was an initial bulky lymph node (≥5 cm), reported in seven patients (35%).

**Figure 3:**
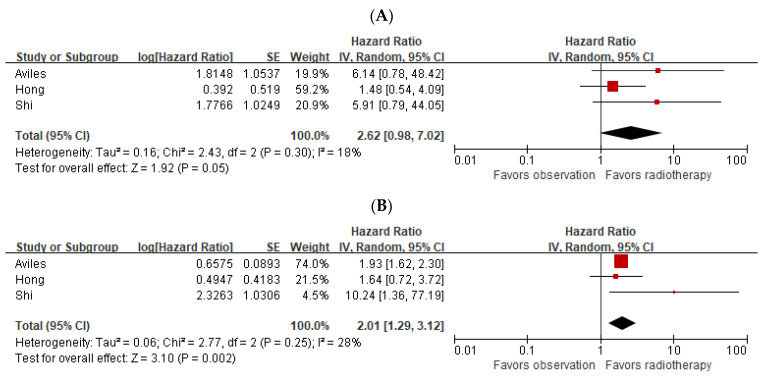
Forest plots of overall survival (**A**) and disease-free survival (**B**) in the subgroup analysis [6,7,12].

We performed a subgroup analysis for studies that included patients who only received irradiation in high-risk areas. The statistical significance of OS was marginal when the subgroup analysis was conducted solely for these studies (*p* = 0.05). The subgroup meta-analysis for DFS revealed a statistically significant difference (HR: 2.01; 95% CI: 1.29–3.12; *p* = 0.002). A forest plot for the subgroup analysis is shown in Figure 3.

The information on treatment-related toxicity was only available in one of the included studies. Dabaja et al. reported two deaths related to toxicity in the R-CHOP alone group and one death in the R-CHOP with RT group [10]. In a study by Shi et al., RT was fairly well tolerated with mild toxicity observed [6]. In a study by Aviles et al. [7], the secondary toxicities induced by RT included grade 1 dermatitis in 21 patients and grade 1 abdominal pain in 3 patients; however, the study did not employ modern RT planning techniques. There were no events of cardiac damage in patients older than 60 who underwent RT. The other three studies provided no analysis or description of treatment-related toxicity.

The patterns of treatment failure in each study are summarized in Table 3. Four studies included descriptions of failure patterns, and two studies evaluated their statistical significance. The overall recurrence patterns were higher in the R-CHOP alone group. Aviles and Hong found that same-site (initially involving lesions) recurrence was more frequent in patients treated with R-CHOP alone [7,12]. More specifically, Aviles et al. found that same-site recurrence was significantly more frequent in the immunochemotherapy alone group (*p* < 0.001) [7]. A multivariate analysis by Hong et al. revealed that consolidative RT was a significant prognostic factor for locoregional-free survival [12]. On the other hand, there were no significant differences between the two groups in distant recurrences at other sites.

## 4. Discussion

The challenges related to consolidative RT in patients with advanced DLBCL include whether to irradiate all pretreatment extent or only bulky areas and which patients may benefit from clinical outcomes. The radiation dose with a consolidative aim for lymphomas is generally lower than that for other solid tumors, so there is less concern about radiation-induced toxicity. However, there is a risk of hematological and neurological toxicity from previously administered systemic chemotherapy. The current clinical opinion suggests a limited efficacy of radiotherapy for palliative treatment in patients with advanced-stage DLBCL [14]. Therefore, it is necessary to establish an indication for treating patients with consolidative RT.

Our meta-analysis of six retrospective studies demonstrated a statistically significant role of consolidative RT in improving both OS and DFS. None of the six studies showed a significant improvement in OS, and only three studies reported a statistically significant improvement in DFS with consolidative RT. In each study, the RT group exhibited an improved survival rate, but it was not statistically significant due to the small sample sizes. The current meta-analysis revealed a statistically significant improvement in the survival rate of advanced-stage DLBCL patients after consolidative RT. In a demographic comparison of the three studies, patients in the consolidative RT group had larger bulky disease or tumor size than those in the no-RT group [6,11,13]. Nevertheless, our meta-analysis indicated that the OS or DFS benefit of consolidative RT may be more significant in patients with comparable disease characteristics. A carefully designed comparative matching study evaluating the role of consolidative RT in low- and intermediate-risk groups would be worthwhile.

Among the six studies included in this meta-analysis, one study irradiated only bulky sites; one study administered radiation to bulky or high-risk areas, such as bones, the head, and the neck with a high risk of recurrence; two studies included all initial extents; one study used a mixed approach; and one study lacked a detailed description. We expected a comprehensive analysis of the results regarding the different radiation fields and indications used in each study. However, detailed information related to treatment indications and outcomes was not available from the data retrieved. Each study lacked specific descriptions of precise treatment indications for radiotherapy. A consensus regarding the radiation field for advanced-stage DLBCL has not been established at many institutions. The subgroup analysis of the three studies that included bulky sites (Aviles > 10 cm; Hong ≥ 5 cm; Shi ≥ 5 cm) as the radiation field showed relatively lower statistical significance in OS than that of the entire study [6,7,12]. Considering the relatively less significant improvement in OS observed in the three studies irradiating only bulky sites, it was expected that employing a radiation field including as much of the initial extent as possible rather than limiting it to specific high-risk lesions would confer a survival gain.

Indeed, Shi et al. analyzed a cohort of patients who underwent involved-site RT targeting bulky sites greater than 5 cm and demonstrated a statistically significant improvement in clinical outcomes, specifically in terms of DFS [6]. Conversely, Dabaja et al. performed a propensity score matching study where they administered RT to all initial disease extents, and their findings revealed no statistically significant differences in DFS [10]. This conflicting evidence underscores the ongoing controversy regarding the optimal irradiation targets for consolidative RT in advanced-stage DLBCL. Further research and consensus are required to determine the most appropriate sites for irradiation to improve clinical outcomes.

All six studies included patients who received at least six cycles of R-CHOP. Although there were concerns regarding hematological and radiation toxicity following chemotherapy, no such toxicities were reported, with a less than 3% incidence of low toxicity observed in two studies. Four studies did not report any toxicity-related adverse events; therefore, toxicity-related analyses were not conducted in this meta-analysis. Consolidative RT was well tolerated for up to 30–36 Gy, with a low incidence of hematological toxicity after R-CHOP [15]. Nevertheless, considering the multifocal nature of DLBCL, which can involve multiple nodal and extranodal sites, the judicious selection of the radiation field based on the specific organs involved is crucial to reduce the potential risks of treatment-related toxicity [16,17].

In the era of intensity-modulated radiation therapy, it has become more feasible to administer RT for head and neck lesions, and the risk of toxicity has decreased. Kwak et al. reported excellent LC with consolidative RT in DLBCL patients with head and neck lesions [18]. In addition, multiple studies have consistently identified bulky disease as a critical prognostic factor for local failure [5,19,20,21,22,23]. Consolidative RT targeting high-risk sites may represent an effective strategy to increase LC and treatment outcomes in patients with DLBCL. This could potentially address the limitation of rituximab, which improved overall survival rates but local recurrence remains the dominant cause of treatment failure [23].

There were various criteria for defining bulky disease in the included studies. The differing definitions of bulky sizes and radiation fields in the analyzed studies were factors that caused heterogeneity in this study. The Mabthera International study suggested that the threshold size for bulky tumors in the rituximab era is likely closer to 10 cm rather than the previously accepted range of 5–10 cm [19]. However, the impact of rituximab on the prognosis of the bulky disease remains uncertain. Bulky tumors are characterized by limited vascular flow, which has been shown to impair drug delivery [24]. Nevertheless, the introduction of rituximab is believed to mitigate this effect. Rituximab’s mechanism of action-targeting CD20 on B-cell lymphoma cells may reduce the impact of bulky tumor burden on drug delivery.

A previous prospective study was conducted to evaluate the efficacy and safety of a combined treatment approach in elderly DLBCL patients with bulky disease [25]. The study revealed a 42% reduction in the utilization of RT without compromising treatment outcomes in PET-negative patients with bulky disease following chemotherapy. However, it is important to note that this analysis represents an interim evaluation, and further investigation and comprehensive analysis of the complete study data will be required to establish more definitive conclusions regarding the overall effectiveness and long-term outcomes of the treatment. Several studies, on the other hand, observed that RT improved the outcome without leading to significant toxicities in the elderly population [26,27]. These controversies highlight the significance of selecting appropriate subjects for RT.

In addition to RT, other consolidative treatments for advanced-stage DLBCL have also been investigated. The SWOG 9704 trial analyzed 4-year PFS rates ranging from 64% to 78% post-autologous stem cell transplantation (ASCT) following rituximab-based induction therapy compared to 50% post-R-CHOP alone for patients with their first remission of diffuse aggressive non-Hodgkin’s lymphoma [28]. Another study reported that high-dose chemotherapy and ASCT with rituximab-based chemotherapy resulted in a 2-year PFS of 72% in patients with intermediate- to high-risk DLBCL, which was higher than the 59% observed in the no-consolidation group [29,30]. The clinical measures for consolidative RT were comparable to those for consolidative ASCT, and further analysis of these two groups is expected to improve the survival rate and provide guidance for optimal adjuvant treatment modalities.

Significant progress has been made recently with respect to the introduction of chimeric antigen receptor (CAR) T-cell therapy as an innovative adjuvant treatment for patients with advanced DLBCL experiencing relapse [31,32,33]. The combination of RT with CAR T-cell therapy shows potential as a therapeutic approach for advanced DLBCL patients. RT delivers targeted high-energy radiation to cancer cells, inducing antigen release and upregulating target antigens in cancer cells, rendering them more susceptible to recognition and elimination by CAR T-cells. This collaborative action creates a more favorable tumor microenvironment. Clinical studies will help elucidate the safety and efficacy of this combination strategy, facilitating its implementation into standard clinical practice.

## 5. Conclusions

In conclusion, a meta-analysis of several retrospective studies has revealed the survival benefit of consolidative RT in patients with advanced-stage DLBCL. For these patients in the rituximab era, additional research on the radiation field or suitable indications for consolidative RT is needed.

## Figures and Tables

**Table 1:** Characteristics of the six studies analyzed in this study.

First Author	Year	Country of Origin	Median Age, Years (Range)	Follow-Up Duration, Months (Range)	Ann Arbor Stage	Total Patients, *n*	RT Group, *n*	No-RT Group, *n*	R-CHOP Cycle	RT Field	RT Target	Radiation Dose, Gy	Cutoff of Bulky Disease, cm
Aviles et al. [7]	2019	Mexico	53 (39–60)	61 (42–104)	III–IV	258	127	131	6	ISRT	Initial bulky site	30	10
Dabaja et al. [10]	2015	US	57 (18–91)	54 (6–128.4)	III–IV (subgroup)	76 (matched pair 1:1)	38	38	6	IFRT	Initial extents	Unknown details	10
Dorth et al. [11]	2012	US	62	56.4 (12–204)	III–IV	79	38	41	6	ISRT	Initial extents	25	7
Hong et al. [12]	2021	Korea	58 (19–81)	39.7 (6.8–125.1)	III–IV	102 (matched pair 1:2)	34	68	6–8	ISRT	Initial bulky (17), H&N (8), bone (6), or testes (3)	30.6	5
Shi et al. [6]	2013	US	59 (20–81)	32.9 (1–151)	III–IV	110	14	96	6	ISRT	Initial extents (7), bulky (7)	30.6	5
Syed et al. [13]	2021	US	59	49.2	III–IV	188	68	120	6	ISRT	Initial extents	30	5

Abbreviations: RT, radiotherapy; R-CHOP, rituximab with cyclophosphamide, doxorubicin, vincristine, and prednisone; ISRT, involved-site radiotherapy; IFRT, involved-field radiotherapy; H&N, head and neck.

**Table 2:** Clinical results of the six studies.

First Author	5-Year OS Rate (%)		5-Year OS HR (95% CI)	*p* Value	5-Year DFS Rate (%)		5-Year DFS HR (95% CI)	*p* Value	5-Year LC Rate (%)	
	RT Group	No-RT Group			RT Group	No-RT Group			RT Group	No-RT Group
Aviles et al. [7]	91.0	56.0	6.14 (0.90–41.10)	0.085	87.0	45.0	1.93 (1.62–2.24)	-	-	-
Dabaja et al. [10]	-	-	1.89 (0.94–3.70)	0.070	-	-	1.30 (0.76–2.17)	0.340	-	-
Dorth et al. [11]	85.0	78.0	2.08 (0.57–7.59)	0.280	85.0	65.0	4.30 (1.30–13.80)	0.014	92	69
Hong et al. [12]	84.7	68.6	1.48 (0.53–4.10)	0.450	75.1	58.2	1.64 (0.72–3.76)	0.237	-	-
Shi et al. [6]	92.3	68.5	5.91 (0.79–44.03)	0.083	85.1	44.2	10.24 (1.36–76.84)	0.024	91.7	48.8
Syed et al. [13]	87.4	72.9	1.82 (0.70–4.7)	0.216	85.9	49.2	4.35 (1.92–10.00)	0.001	91.9	54.9

Abbreviations: OS, overall survival; DFS, disease-free survival; LC, local control; RT, radiotherapy; HR, hazard ratio; CI, confidence interval.

**Table 3:** Pattern of recurrence in available studies.

	R-CHOP + RT			R-CHOP Alone			*p* Value		
	Same Site Recurrence (%)	Other Site Recurrence (%)	Both (%)	Same Site Recurrence (%)	Other Site Recurrence (%)	Both (%)	Same Site Recurrence (%)	Other Site Recurrence (%)	Both (%)
Aviles et al. [7]	0	7	9	18	5	36	<0.001	0.66	<0.001
Dabaja et al. [10]	7.7	8.8	7.2	22.6	17.1	13.3	-	-	-
Dorth et al. [11]	2.6	2.6	5.3	7.3	7.3	17.1	-	-	-
Hong et al. [12]	5.9	5.9	11.8	20.6	7.4	8.8	0.055	0.783	0.639

Abbreviations: R-CHOP, rituximab with cyclophosphamide, doxorubicin, vincristine, and prednisone; RT, radiotherapy.

## Data Availability

Not applicable.

## Supplementary Materials

The following supporting information can be downloaded at: https://www.mdpi.com/article/10.3390/cancers15153940/s1, Table S1: The Newcastle-Ottawa Scale scoring system; Figure S1. Funnel plots of overall survival (A) and disease-free survival (B) in the analyzed studies.
