# Supplementary material for: Consolidative Radiotherapy after Complete Remission following R-CHOP Immunochemotherapy in Stage III–IV Diffuse Large B-Cell Lymphoma Patients: A Systematic Review and Meta-Analysis
Source: Cancers (Basel). 2023 Aug 2;15(15):3940. doi: 10.3390/cancers15153940 (PMC10417435; doi:10.3390/cancers15153940)

**Supplementary Materials Table S1.** The Newcastle‒Ottawa Scale scoring system

|  | Selection | | | | Comparability | Outcome | | | Overall score  (9 to be full) |
| --- | --- | --- | --- | --- | --- | --- | --- | --- | --- |
|  | Representativeness of the exposed cohort | Selection of the nonexposed cohort | Ascertainment of exposure | Outcome of interest was not present at start of study | Comparability of cohorts on the basis of the design or analysis | Assessment of outcome | Was follow-up long enough for outcomes to occur | Adequacy of follow-up of cohorts |  |
| Aviles et al. [7] | 1 | 1 | 1 | 1 | 1 | 1 | 1 | 1 | 8 |
| Dabaja et al. [10] | 1 | 1 | 1 | 1 | 1 | 1 | 1 | 1 | 8 |
| Dorth et al. [11] | 1 | 1 | 1 | 1 | 0 | 1 | 1 | 1 | 7 |
| Hong et al. [12] | 1 | 1 | 1 | 1 | 1 | 1 | 1 | 1 | 8 |
| Shi et al. [6] | 1 | 1 | 1 | 1 | 0 | 1 | 1 | 1 | 7 |
| Syed et al. [13] | 1 | 1 | 1 | 1 | 0 | 1 | 1 | 1 | 7 |

**Supplementary Materials Figure S1.** Funnel plots of overall survival (A) and disease-free survival (B) in the analyzed studies

1. (B)


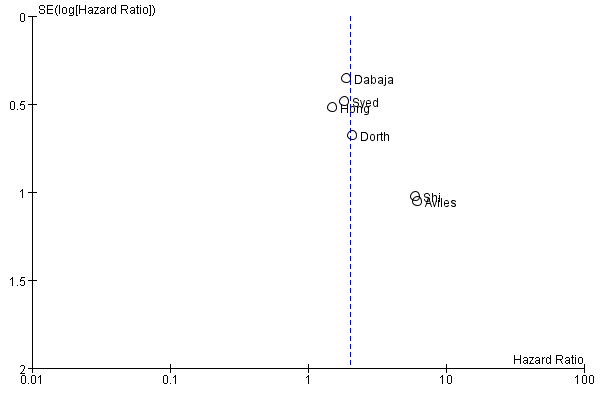

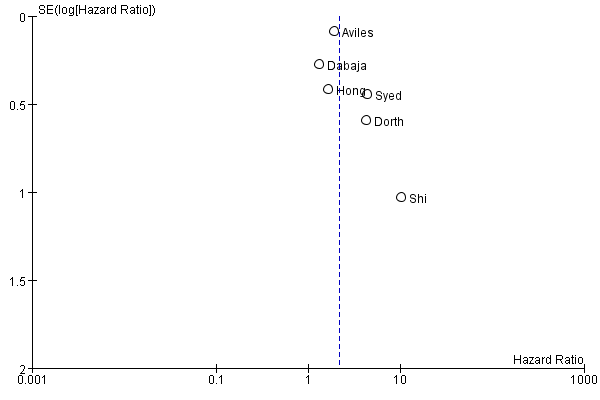

Supplement: Supplementary file 1 [file cancers-15-03940-s001.zip › cancers-2502044-supplementary.docx]
